# Quantum asymmetric key crypto scheme using Grover iteration

**DOI:** 10.1038/s41598-023-30860-0

**Published:** 2023-03-07

**Authors:** Chun Seok Yoon, Chang Ho Hong, Min Sung Kang, Ji-Woong Choi, Hyung Jin Yang

**Affiliations:** 1 Department of Physics, Korea University, Sejong, 30019 South Korea; 2 Institute of Convergence Technology KT R&D Center, 151 Taebong-Ro, Seoul, 06763 Republic of Korea; 3 The Affiliated Institute of Electronics and Telecommunications Research Institute, P.O. Box 1, Yuseong, Daejeon 34188 Republic of Korea; 4 Korean Intellectual Property Office (KIPO), Government Complex Daejeon Building 4, 189, Cheongsa-Ro, Seogu, Daejeon, 35208 Republic of Korea; 5 Center for Quantum Information, Korea Institute of Science and Technology (KIST), Seoul, 02792 Republic of Korea

**Keywords:** Quantum information, Information technology

## Abstract

Here, we propose a quantum asymmetric key cryptography scheme using Grover’s quantum search algorithm. In the proposed scheme, Alice generates a pair of public and private keys, keeps the private keys safe, and only discloses public keys to the outside. Bob uses Alice's public key to send a secret message to Alice and Alice uses her private key to decrypt the secret message. Furthermore, we discuss the safety of quantum asymmetric key encryption techniques based on quantum mechanical properties.

## Introduction

One of the most important advancements in modern cryptographic systems is the development of asymmetric-key cryptography algorithms. These systems allow us to solve the problems of existing symmetric key cryptography algorithms, such as sharing of secure keys between users, management of keys that increase significantly with the number of participants, and problem of not being able to perform authentication. Asymmetric key cryptography systems have become extremely useful for implementing various cryptographic services such as authentication and signatures^1–3^.

In the field of quantum cryptography, which has recently been studied worldwide, various protocols such as quantum direct communication and quantum authentication/signature as well as quantum key distribution, which has entered the commercialization stage through a pilot network, are being developed^4–13^. However, most quantum authentication/signature protocols are being developed based on symmetric key encryption techniques owing to the absence of an efficient quantum asymmetric key crypto scheme. Hence, various tools were utilized to overcome the limitations of symmetry key techniques, which resulted in exposing the disadvantages of protocols being assisted by classical elements or via complex implementation^14–21^.

Furthermore, quantum asymmetric key crypto schemes are studied on a smaller scale when compared to other protocols; however, they have a number of similar shortcomings, such as being aided by classical elements, application of symmetric key techniques, or having a structure that makes it difficult to apply the actual public key concept using a quantum entanglement state^22–36^.

In this paper, an efficient quantum asymmetric key cryptography scheme based on Grover’s algorithm has been proposed^37,38^. The asymmetric key cryptography systems used in modern cryptographic systems are analyzed, and the basic structure of Grover’s algorithm is discussed in Section “Asymmetric cipher and Grover algorithm”. Subsequently, a quantum asymmetric key cryptography system using Grover’s algorithm is proposed in section "Quantum asymmetric key cipher scheme using Grover algorithm". Finally, the security of the system is analyzed in Section "Security analysis".

## Asymmetric cipher and grover algorithm

### Asymmetric cipher

The specific method of the asymmetric key cryptography system was first introduced in the study, “New directions in cryptography” by Whitfield Diffie and Martin Hellman at Stanford University in 1976. Subsequently, in 1978, Ronald L. Rivest, Adi Shamir, and Leonard Adleman (RSA) at MIT implemented a public key cryptographic system, which is known as the RSA public key cryptography algorithm that is used extensively at present^39,40^.

An asymmetric key cryptographic system is based on a trapdoor one-way function. Here, a one-way function refers to a problem in which the calculation in one direction is simple, whereas the calculation of an inverse function (e.g., a hash function) is impossible. A function with a trapdoor, which facilitates the calculation of an inverse function based on certain hints, is known as a trapdoor one-way function. A cryptographic system that allows only people who possess specific information to perform decryption easily based on this function is known as an asymmetric key cryptography system^1–3^.

**Figure 1:**
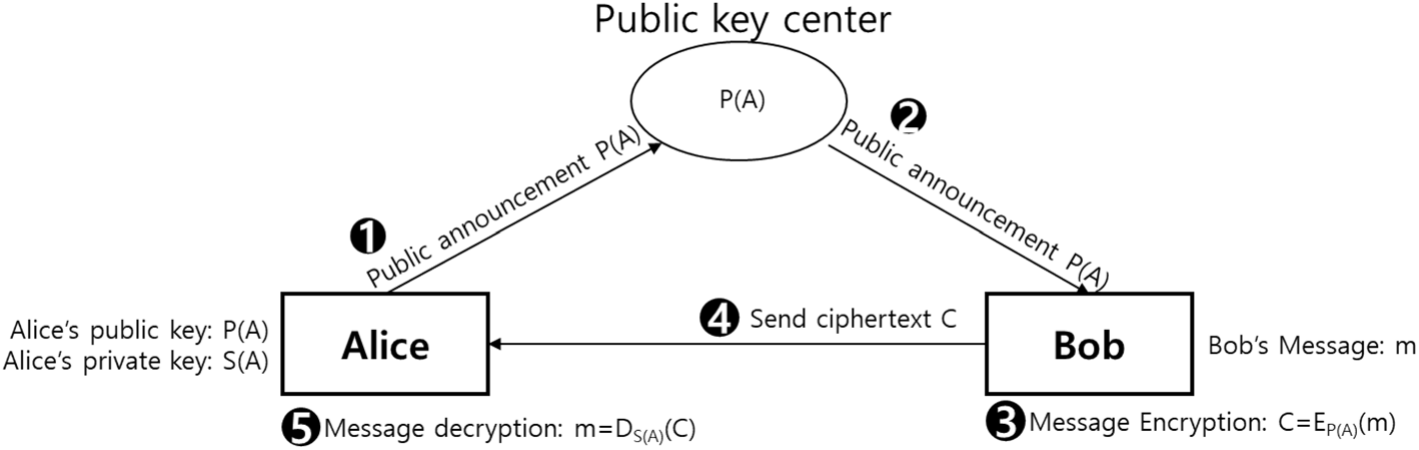
Asymmetric cryptography system uses an encryption key that consists of a public key and private key pair. Security in asymmetric key encryption systems relies on managing private keys without exposing them to the outside world. In contrast, public keys can be released to others. Anyone with a public key can create and send a secret message at any time, whereas someone with a private key can open the secret message at any time. In this figure, Bob, who shares Alice's public key P(A), encrypts the message m he wants to send to Alice, and Alice decrypts the message using her private key S(A).

As depicted in Fig. 1, Alice generates a public key P(A) and private key S(A) for herself. The public key is disclosed so that anyone can use it. Bob uses Alice’s public key to encrypt a message to be sent to Alice. The encrypted message can be decrypted using the private key that Alice possesses. Bob can securely send a message that can be viewed only by Alice, but he is not required to have a common key with Alice in advance. This is known as asymmetric key cryptographic system.

### Grover iteration

Grover’s algorithm, also known as the quantum search algorithm, determines whether the information to be obtained exists in a database. In previous algorithms pertaining to information search schemes, the time complexity for obtaining the desired data in a database composed of N unsorted data is $O\left(N\right)$. However, the quantum search algorithm proposed by Grover in 1997 uses the quantum entanglement phenomenon to obtain the desired data faster when compared to previous methods, and the time complexity is expressed as $O\left(\sqrt{N}\right)$^7,37,38^.

The operating principle of Grover’s algorithm is as follows:

A database consisting of unsorted data, which contain the desired data is expressed as follows:

1 $$\left| M \right\rangle = \left( \frac{1}{N} \right)\sum\nolimits_{i = 0}^{N - 1} {\sum\nolimits_{j = 0}^{N - 1} {\left| {i,\;j} \right\rangle } }$$

The data that you want to find in a database composed of unsorted data is as follows:

2 $$\left| K \right\rangle = {\left| {i_{0},\;j_{0}} \right\rangle }$$

The operators required to find the desired data in the database are as follows:

3 $$U_{S} = I - N\left| K \right\rangle \left\langle K \right|,\;U_{V} = C\left| M \right\rangle \left\langle M \right| - I$$

Extraction of the desired data from Eq. (2) by applying Eq. (3) into the database expressed in Eq. (1) is as follows:

4 $$\begin{aligned} U_{V} U_{S} \left| M \right\rangle = & \left( {C\left| M \right\rangle \left\langle M \right| - I} \right)\left( {I - N\left| K \right\rangle \left\langle K \right|} \right)\left| M \right\rangle \\ = & \left( {C\left| M \right\rangle \left\langle M \right| - I} \right)\left( {\left| M \right\rangle - \left| K \right\rangle } \right) \\ = & C\left| M \right\rangle - \left| M \right\rangle - \left( {\frac{1}{{\left( {N - 1} \right)}}} \right)\left| M \right\rangle + \left| K \right\rangle = \left| K \right\rangle \\ \end{aligned}$$

$$\because \langle K \left| M \right\rangle = \frac{1}{N}, \;C = \frac{N}{{\left( {N - 1} \right)}}$$

Equation (4) shows that the desired data $\left|K\right.\rangle$ can be obtained using Eqs. (1, 2, 3) in the database set $\left|M\right.\rangle$. Here, database $\left|M\right.\rangle$ exists in an entangled state. The attacker will be unaware of the exact state of the quantum bit in entangled state because of the singularity of measurement and collapse of quantum state.

In this study, we used the properties of quantum mechanics and the abovementioned advantage to propose a quantum asymmetric cryptography system using the major operation associated with Eq. (4), which is used in Grover’s algorithm.

## Quantum asymmetric key cipher scheme using Grover algorithm

**Figure 2:**
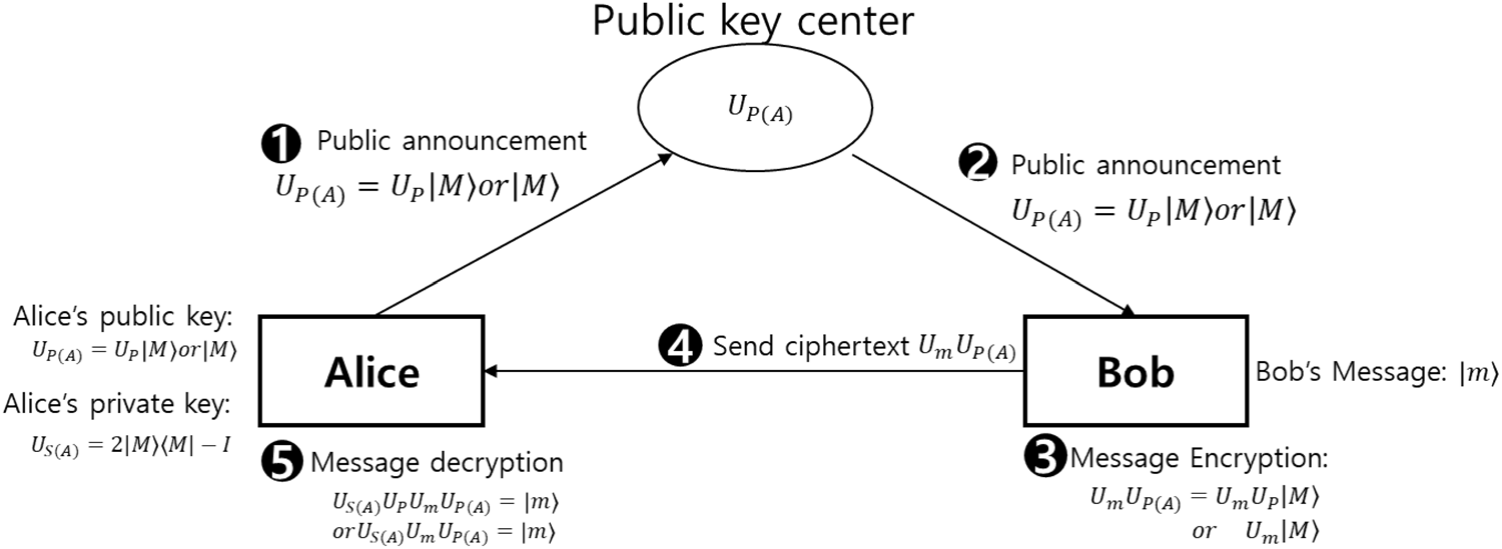
Quantum asymmetric key cipher scheme: This figure is a quantum version of the classical asymmetric key cipher scheme depicted in Fig. 1.

As depicted in Fig. 2, quantum asymmetric key cryptography scheme proposed herein consists of the following phases: a phase in which Alice creates a public key and private key, similar to the modern cryptographic system; a phase where Bob uses Alice’s public key to send a ciphertext; a phase in which the ciphertext is decrypted.

### Preparation phase

#### P1.

 Alice creates a state $\left|M\right.\rangle$ that is only known to her.

5 $$\left| M \right\rangle = \left| a \right\rangle \otimes \left| b \right\rangle$$

(Here, $\left|a\right.\rangle$ and $\left|b\right.\rangle$ are selected arbitrarily from $\left|+\right.\rangle$ and $\left|-\right.\rangle$, respectively.)

#### P2.

 Alice determines an arbitrary two-bit key ${K}_{AP}$, that is used as the material of the public key. Subsequently, the following operator is created:

6 $$U_{P} = I - 2\left| {K_{AP} } \right\rangle \left\langle {K_{AP} } \right|$$

#### P3.

 Alice uses $\left|M\right.\rangle$ and ${U}_{P}$ to create a public key ${U}_{P(A)}$ state and sends it to the public key management center. Here, information exposure can be prevented by randomly selecting whether the operator ${U}_{P}$ is applied.

7 $$\begin{gathered} U_{P\left( A \right)} = U_{P} \left| M \right\rangle = \left( {I - 2\left| {K_{AP} } \right\rangle \left\langle {K_{AP} } \right|} \right)\left( {\left| a \right\rangle \otimes \left| b \right\rangle } \right) \hfill \\ or \hfill \\ U_{P\left( A \right)} = \left| M \right\rangle = \left( {\left| a \right\rangle \otimes \left| b \right\rangle } \right) \hfill \\ \end{gathered}$$

### Encryption phase

#### E1.

 Bob creates the following operator using message $\left|m\right.\rangle$ to be sent to Alice.

8 $$U_{m} = I - 2\left| m \right\rangle \left\langle m \right|$$

#### E2.

 Bob creates the following quantum state using the operator of the message that he wishes to send using Alice’s public key, ${U}_{P(A)}$.

9 $$\begin{gathered} U_{m} U_{P\left( A \right)} = U_{m} U_{P} \left| M \right\rangle = \left( {I - 2\left| m \right\rangle \left\langle m \right|} \right)\left( {I - 2\left| {K_{AP} } \right\rangle \left\langle {K_{AP} } \right|} \right)\left( {\left| a \right\rangle \otimes \left| b \right\rangle } \right) \hfill \\ or \hfill \\ U_{m} U_{P\left( A \right)} = U_{m} \left| M \right\rangle = \left( {I - 2\left| m \right\rangle \left\langle m \right|} \right)\left( {\left| a \right\rangle \otimes \left| b \right\rangle } \right) \hfill \\ \end{gathered}$$

Equation (9) shows the encrypted state of message $\left|m\right.\rangle$ that Bob wishes to send by using Alice’s public key. Bob sends an encrypted message qubit to Alice.

### Decryption phase

#### D1.

 In the message that Alice receives from Bob, if $\left|{U}_{P}\right.\rangle$ exists in the public key that Alice created, then operator $\left|{U}_{P}\right.\rangle$ is re-applied.

10 $$\begin{aligned} U_{P} U_{m} U_{{P\left( A \right)}} = & U_{P} U_{m} U_{P} \left| M \right\rangle \\ = & \left( {{\text{I}} - 2\left| {{\text{K}}_{{{\text{AP}}}} } \right\rangle \left\langle {{\text{K}}_{{{\text{AP}}}} } \right|} \right)\left( {{\text{I}} - 2\left| m \right\rangle \left\langle m \right|} \right)\left( {{\text{I}} - 2\left| {{\text{K}}_{{{\text{AP}}}} } \right\rangle \left\langle {{\text{K}}_{{{\text{AP}}}} } \right|} \right)\left( {\left| a \right\rangle \otimes \left| b \right\rangle } \right) \\ = & \left( {{\text{I}} - 2\left| {{\text{K}}_{{{\text{AP}}}} } \right\rangle \left\langle {{\text{K}}_{{{\text{AP}}}} } \right|} \right)\left( {\left| M \right\rangle - \left| {{\text{K}}_{{{\text{AP}}}} } \right\rangle + \left( {2\delta _{{m,~K_{{AP}} }} - 1} \right)\left| m \right\rangle } \right) \\ = & \left( {\left| M \right\rangle - \left| {{\text{K}}_{{{\text{AP}}}} } \right\rangle } \right) - \left( {\left| {{\text{K}}_{{{\text{AP}}}} } \right\rangle - 2\left| {{\text{K}}_{{{\text{AP}}}} } \right\rangle } \right) + \left( {2\delta _{{m,~K_{{AP}} }} - 1} \right)\left( {\left| m \right\rangle - \left( {2\delta _{{m,~K_{{AP}} }} } \right)\left| m \right\rangle } \right) \\ = & \left( {\left| M \right\rangle - \left| {{\text{K}}_{{{\text{AP}}}} } \right\rangle } \right) - \left( {\left| {{\text{K}}_{{{\text{AP}}}} } \right\rangle - 2\left| {{\text{K}}_{{{\text{AP}}}} } \right\rangle } \right) + \left( {1 - 2\delta _{{m,~K_{{AP}} }} } \right)\left( {2\delta _{{m,~K_{{AP}} }} - 1} \right)\left| m \right\rangle \\ = & \left| M \right\rangle - \left( {2\delta _{{m,~K_{{AP}} }} - 1} \right)^{2} \left| m \right\rangle = \left| M \right\rangle - \left| m \right\rangle \\ \end{aligned}$$

#### D2.

 Alice uses $\left|M\right.\rangle$ to create her own private key ${U}_{S(A)}$.

11 $$U_{S\left( A \right)} = 2\left| M \right\rangle \left\langle M \right| - I$$

#### D3.

 Alice applies her private key operator ${U}_{S(A)}$ to the quantum state created using Eq. (10) and obtains the message $\left|m\right.\rangle$ that Bob wishes to send to her.

$$\begin{aligned} U_{S\left( A \right)} U_{P} U_{m} U_{P\left( A \right)} = & U_{S\left( A \right)} U_{P} U_{m} U_{P} \left| M \right\rangle \\ = & \left( {2\left| M \right\rangle \left\langle M \right| - I} \right)\left( {I - 2\left| {K_{AP} } \right\rangle \left\langle {K_{AP} } \right|} \right)\left( {I - 2\left| m \right\rangle \left\langle m \right|} \right) \\ & \left( {I - 2\left| {K_{AP} } \right\rangle \left\langle {K_{AP} } \right|} \right)\left( {\left| a \right\rangle \otimes \left| b \right\rangle } \right) \\ = & \left| m \right\rangle \\ \end{aligned}$$

12 $$\begin{aligned} or \\ U_{S\left( A \right)} U_{m} U_{P\left( A \right)} = & U_{S\left( A \right)} U_{m} \left| M \right\rangle \\ = & \left( {2\left| M \right\rangle \left\langle M \right| - I} \right)\left( {I - 2\left| m \right\rangle \left\langle m \right|} \right)\left( {\left| a \right\rangle \otimes \left| b \right\rangle } \right) \\ = & \left| m \right\rangle \\ \end{aligned}$$

## Security analysis

### Confidentiality

The component that requires confidentiality in the proposed scheme is the message $\left|m\right.\rangle$ that Bob wishes to send to Alice. This message must not be revealed to anyone except Bob, who sends the message, and Alice, who receives it. There is a possibility for Eve to attack once, while message $\left|m\right.\rangle$ is sent from Bob to Alice.

First, the qubit that Eve uses is in an entangled quantum state based on Eq. (9). If Eve measures the qubit, the quantum state collapses and the state prior to the collapse is not known. Furthermore, it cannot be measured accurately without knowing $\left|{U}_{P}\right.\rangle$ and $\left|{U}_{S(A)}\right.\rangle$, that is, information required for decryption. In other words, Eve’s measurements cannot accurately determine the entangled quantum state.

Second, methods for attacking quantum cryptography include stealing information by creating an entangled state using a CNOT operator. However, in the entangled quantum state, as expressed by Eq. (9), the information that can be obtained based on the attack method using the CNOT operator is the quantum state, which is a component of the public key expressed in Eq. (7). If this is explained on the basis of the two-bit quantum state expressed in Eq. (5), only the state of $\left|00\right.\rangle ,\left|01\right.\rangle ,\left|10\right.\rangle , \mathrm{and }\left|11\right.\rangle$ will be known, and the preceding phases will not be known. However, only when the phases are known, can Eve decipher message $\left|m\right.\rangle$. In other words, if Eve steals the quantum state of the encrypted message being sent, then message $\left|m\right.\rangle$ will not be known.

### Kerchhoffs’ principle

A cryptographic algorithm must provide security even if all components excluding the keys used in the algorithm are disclosed.

Based on Kerchhoffs’ principle, the cryptographic algorithm should provide security even if all components excluding the private key used are exposed to the attacker. In the proposed algorithm, security is ensured, unless Alice’s key material $\left|M\right.\rangle$ is exposed. Even if Eve steals Alice’s public keys ${U}_{P}\left|M\right.\rangle$ and ${U}_{m}{U}_{P}\left|M\right.\rangle$ which Bob sends to Alice, an accurate measurement cannot be performed. Therefore, Eve does not know the important key material $\left|M\right.\rangle$ and message $\left|m\right.\rangle$ that Bob wishes to send. In other words, even if Eve knows all the stages of the algorithm, it is impossible to obtain relevant information.

### Shor’s algorithm

It has been reported that Shor’s algorithm^41,42^, which is a typical quantum algorithm, can effectively unravel modern public-key cryptographic algorithms. By effectively factoring integers, the possibility of performing critical attacks on modern cryptographic systems via quantum computers has been demonstrated. However, attacks by Shor’s algorithm are threats to modern cryptographic systems that rely on computational complexity; however, they do not pose a specific threat to quantum cryptographic systems that are based on the characteristics of quantum mechanics.

Because the proposed quantum public-key cryptographic scheme guarantees security based on the characteristics of quantum mechanics, it is no relevant to attacks by Shor’s algorithm. In other words, the proposed scheme is safe from attacks using Shor’s algorithm.

### Comparison of protocol efficiencies

We can compare the efficiency of our scheme with that of existing schemes in terms of the required quantum sources and qubit efficiency.

The qubit efficiency can be defined as follows:

$$Qubit \,efficiency = \frac{c}{q}$$

In this definition, c denotes the total number of classical message bits and q denotes the total number of qubits.

As listed in Table 1, our quantum asymmetric key cryptography scheme is more efficient when compared to other schemes.

**Table 1:** Comparison of the efficiency of our protocol with that of other quantum asymmetric key crypto schemes^28,34,36^.

	^28^	^34^	^36^	Proposed scheme
Quantum source	Single qubit	Mixed state	Bell state	2N single qubits
Key spaces	Big	Big	Small	Small
Security of private key	One way security	One way security	One way security	One way security
Qubit efficiency	1	< 50%	50%	1
Decryption error	No	Yes	No	No

## Conclusion

Here, we proposed a quantum asymmetric key cryptographic scheme using Grover’s algorithm. In the proposed scheme, Alice uses the public and private keys that she created, and only the public key is disclosed to the outside. Furthermore, Bob can send private messages that are only viewable by Alice without sharing any key with Alice. Even if Eve knows everything about the algorithm and steals the qubit being transmitted, she will not know the content of the private message or Alice’s private key. Our proposed quantum asymmetric key cryptography scheme is safe from attacks because its security is based on entanglement, measurement, and collapse, which are the characteristics of quantum mechanics.

### Future work:

 Contrary to what we have proposed in this paper, if the message is encrypted with the private key and the encrypted message is decrypted with the public key, it is expected to be used as a quantum authentication/signature protocol with integrity and non-repudiation. Discussions on this topic are considered for future works.

## Data Availability

All data generated or analyzed during this study are included in this published article.
